# Emerging SARS-CoV-2 variants of concern potentially expand host range to chickens: insights from AXL, NRP1 and ACE2 receptors

**DOI:** 10.1186/s12985-023-02123-x

**Published:** 2023-08-29

**Authors:** Mengyue Lei, Ying Ma, Hongli Chen, Pu Huang, Jing Sun, Xu Wang, Qiangming Sun, Yunzhang Hu, Jiandong Shi

**Affiliations:** 1 Yunnan Provincial Key Laboratory of Vector-borne Diseases Control and Research, Institute of Medical Biology, Chinese Academy of Medical Sciences and Peking Union Medical College, 935 Jiaoling Road, Kunming, 650118 Yunnan Province China; 2 National Kunming High-level Biosafety Primate Research Center, Institute of Medical Biology, Chinese Academy of Medical Sciences and Peking Union Medical College, Yunnan, China; 3 Kunming Medical University, Kunming, Yunnan China

**Keywords:** SARS-CoV-2 variants, Chickens, ACE2, AXL, NRP1, Cross-species transmission

## Abstract

**Background:**

The possibilities of cross-species transmission of SARS-CoV-2 variants of concern (VOCs) between humans and poultry species are unknown. The analysis of the structure of receptor was used to investigate the potential of emerging SARS-CoV-2 VOCs to expand species tropism to chickens based on the interaction between Spike (S) protein and tyrosine kinase receptor UFO (AXL), angiotensin-converting enzyme 2 (ACE2), and neuropilin 1 (NRP1) with substantial public health importance.

**Methods:**

The structural and genetic alignment and surface potential analysis of the amino acid (aa) in ACE2, AXL, and NRP1 in human, hamster, mouse, mink, ferret, rhesus monkey and chickens were performed by Swiss-Model and pymol software. The critical aa sites that determined the susceptibility of the SARS-CoV-2 to the host were screened by aligning the residues interfacing with the N-terminal domain (NTD) or receptor-binding domain (RBD) of Spike protein.

**Results:**

The binding modes of chickens AXL and ACE2 to S protein are similar to that of the ferret. The spatial structure and electrostatic surface potential of NRP1 showed that SARS-CoV-2 VOCs could not invade chickens through NRP1 easily.

**Conclusion:**

These results suggested that emerging SARS-CoV-2 VOCs potentially expand the host range to chickens mainly through ACE2 and AXL receptors, while NRP1 receptor may rarely participate in the future epidemic of coronavirus disease 2019 in chickens.

## Background

Since its discovery in 2019, severe acute respiratory syndrome coronavirus 2 (SARS-Cov-2) has spread rapidly, posing a huge threat to global public health safety and affecting population life expectancy worldwide. With the evolution and prevalence of viruses, new variants are constantly emerging. Notably, various animals that have intersected closely with humans have been found to be infected with the virus. This ability to spread across species originates primarily from mutations in the spike protein of viruses and host receptors [1][2][3]. However, the potential of SARS-CoV-2 VOCs spread in poultry is yet unknown.

Recent studies have shown that the receptor binding domain (RBD) of S protein of SARS-CoV-2 can interact with ACE2 in multiple species, including monkeys, horses, and sheep but cannot bind the ACE2 of mice [4]. The original strain of SARS-CoV-2 cannot infect the ordinary laboratory mouse models because of differences between mouse and human ACE2 receptors at key amino acid sites for viral activity. Subsequently, the beta variant produced by the virus mutation can infect laboratory mice by intranasal instillation [5], suggesting that SARS-CoV-2 VOCs acquired cross-species infectivity during evolution. A recent study suggested that the interspecific evolutionary trajectory of the Omicron outbreak between human and mice [6]. The new mutations at key amino acid sites in its spike protein may cause new future variants to gain stronger infectious capacity, intensify human-animal-environment transmission, and initiate a new public health emergency. Therefore, predicting the potential host of emerging SARS-CoV-2 variants would provide new insights for the prevention and control of the virus and help to understand the mechanism of cross-species transmission of SARS-CoV-2.

Angiotensin-converting enzyme 2 (ACE2) is a major receptor for SARS-CoV-2 entry into host cells. However, ACE2 has low/medium expression in the human respiratory system, indicating that in addition to ACE2, other host factors may play a major role in mediating the entry of SARS-CoV-2 into host cells. Tyrosine-protein kinase receptor UFO (AXL) [7] and neuropilin-1 (NRP1) [8], a newly discovered viral receptor of SARS-CoV-2, promote the entry of SARS-CoV-2 into host cells and infection. The key to cross-species transmission of SARS-CoV-2 is the degree of affinity with the receptor, which expands the potential viral reservoir with the discovery of new SARS-CoV-2 receptors and is a known target of cross-species transmission.

Chickens are livestock with great economic value in animal husbandry, is closely related to human animal husbandry production and life, and its meat and eggs are the primary sources of protein for humans. Currently, the *Gammacoronavirus* of the coronaviruses family can infect chickens, causing infectious bronchitis[9], although chickens are less susceptible to SARS-CoV-2 original strain in both natural and laboratory immunizations [10][11]. Birds are susceptible to Gamma and Delta coronavirus subfamilies, and studies have shown that the Porcine Delta-coronavirus Diease(PDCoV) in pigs, a virus that uses host aminopeptidase N as an entry receptor, can infect a wide range of species, including human and chickens cells [12]. However, whether SARS-CoV-2 VOCs potentially spreads to chickens across species is yet unknown. If new variants of SARS-CoV-2 in the future break through the species barrier and gain the ability to infect chickens, they will evolve in large flocks, causing a new pandemic that would be a global public health-threatening.

In order to speculate whether SARS-CoV-2 VOCs can be transmitted to chickens, we compared the differences in the key amino acid residues of AXL, NRP1, and ACE2 receptors between human, rhesus, hamster, mouse, ferret, mink, and chickens and the surface potential of the protein to bind to SARS-CoV-2.

## Methods

### Data retrieval

The FASTA sequences of the three strains were obtained from GenBank: Wuhan-Hu-1 (GenBank accession no.**YP_009724390**), Delta variant (GenBank accession no. **QYM88683**), and Omicron variant (GenBank accession no. **UFS23237**). The FASTA sequences of AXL, NRP1, and ACE2 receptors of human, rhesus, hamster, mouse, ferret, mink, chickens were also obtained from GenBank as follows: AXL: human (GenBank accession no. **NP_068713.2**), rhesus (GenBank accession no. **XP_028695606.1**), hamster (GenBank accession no. **XP_035292416.1**), mouse (GenBank accession no. **XP_006540052.1**), ferret (GenBank accession no. **XP_004776133.1**), mink (GenBank accession no. **XP_044113292.1**), and chickens (GenBank accession no. **NP_989958.2**); NRP1: human (GenBank accession no. **XP_006717584.1**), rhesus (GenBank accession no.**NP_001252745.1**), hamster (GenBank accession no. **XP_040590606**), mouse (GenBank accession no. **NP_033491**), ferret (GenBank accession no. **XP_004774343.2**), mink (GenBank accession no. **XP_044082878.1**), and Gallus (GenBank accession no. **NP_990113.1**); ACE2: human (GenBank accession no. **NP_001358344.1**), rhesus (GenBank accession no. **NP_001129168.1**), hamster (GenBank accession no. **XP_003503283.1**),mouse (GenBank accession no. **NP_001123985.1**), ferret (GenBank accession no. **NP_001297119.1**), mink (GenBank accession no. **XP_044091953**), and chickens and space (GenBank accession no. **XP_040517014.1**). The AXL, NRP1, and ACE2 protein models of rhesus, hamster, mouse, ferret, mink, and chickens were derived from three protein models with PDB numbers 4yfg, 7m0r, and 6m18, respectively, in the human protein database (PDB).

### Sequence analysis of AXL, NRP1, and ACE2 proteins

The FASTA sequences of receptor proteins of seven species and three virus strains from GenBank were compared using clusterW alignment software from the European Bioinformatics Institute, and the results were displayed using https://espript.ibcp.fr/ESPript/cgi-bin/ESPript.cgi [13]. The extracellular intracellular analysis of proteins used topcons (https://topcons.cbr.su.se/) to make predictions [14][15].

### Structure simulation of receptor-S complex

The NCBI structure server ( https://www.ncbi.nlm.nih.gov/Structure/cdd/wrpsb.cgi) was utilized to analyze the protein domains. The secondary and tertiary structures of AXL, NRP1, and ACE2 of humans are modeled by Swiss-Model (https://swissmodel.expasy.org/); also, AXL and NRP1 of the other species are analyzed by Swiss-Model based on the structures of humans [16][17]. The surface potential diagram of the interface zone of the protein and the surface contact sites of the related and S proteins of each species were identified using pymol software. Then, the electrostatic potential energy map of the displayed protein was calculated.

## Results

### Structures of SARS-CoV-2 S protein complexed with AXL, NRP1, and ACE2 of chickens and six mammal species

**Fig. 1:**
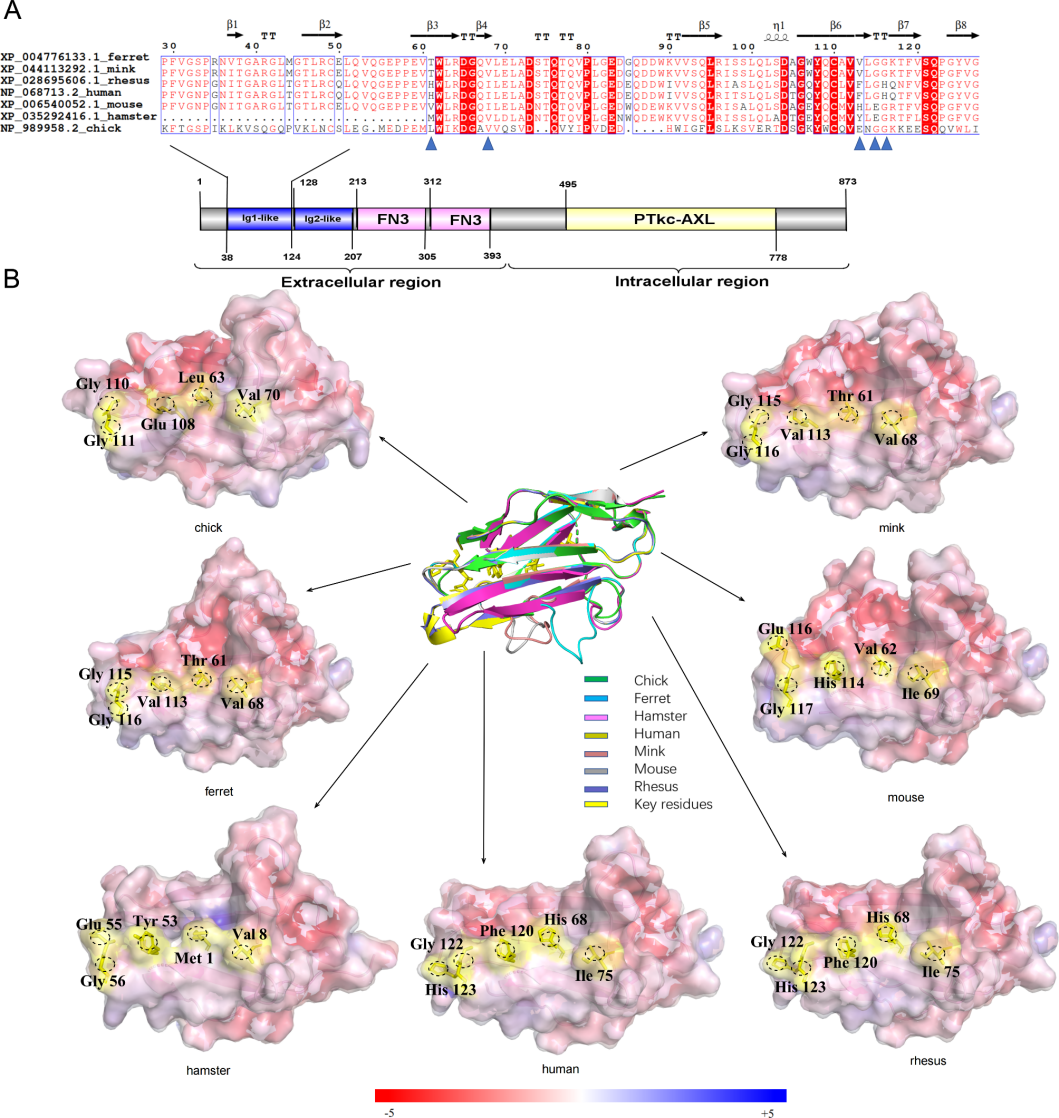
Alignment and surface potential analysis of crucial amino acids in AXL proteins. (**A**) Comparative analysis of the residues of AXLs at the interface binding to the spike of SARS-CoV-2 from human (GenBank accession no. NP_068713.2), rhesus (GenBank accession no. XP_028695606.1), hamster (GenBank accession no. XP_035292416.1), mouse (GenBank accession no. XP_006540052.1), ferret (GenBank accession no. XP_004776133. 1), mink (GenBank accession no. XP_044113292.1), and chickens (GenBank accession no. NP_989958. 2). ALX residues at position 61, 68, 113, 115, and 116 are marked in blue triangles. (**B**) Surface diagram of interface zone of AXLs. The structural superposition of the AXL region 29–127 from human (yellow, PDB code 4yfg), rhesus (violet), hamster (purple), mouse (gray), ferret (cyan), mink (red), and chickens (green). The AXL structures of chickens, rhesus, hamster, mouse, ferret, and mink were constructed using the homology models of human AXL (PDB code 4yfg) as the templates by SWISS-MODEL (https://swissmodel.expasy.org/). The five key differential residues of AXL interacting with spike protein of SARS-CoV-2 are represented by yellow sticks in the structural superposition, the black dash line circled key residues in the potential surface diagram. The electrostatic potential color range is -/+5

**Fig. 2:**
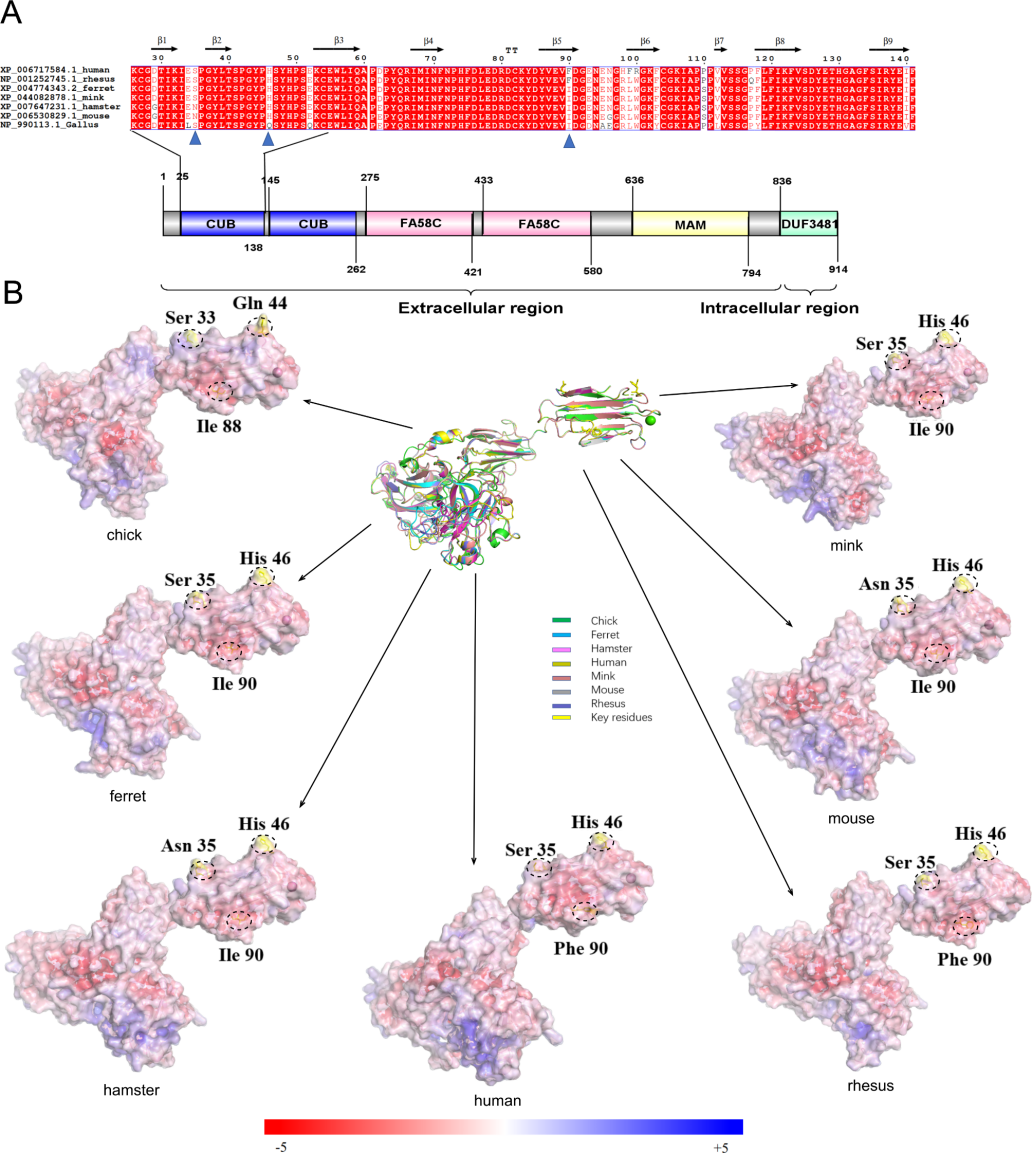
Alignment and surface potential analysis of crucial amino acids in NRP1 proteins. (**A**) Comparative analysis of the residues of NRP1s at the interface binding to the spike protein of SARS-CoV-2 from human (GenBank accession no. XP_006717584.1), rhesus (GenBank accession no. NP_001252745.1), hamster (GenBank accession no. XP_040590606), mouse (GenBank accession no. NP_033491), ferret (GenBank accession no. XP_004774343.2), mink (GenBank accession no. XP_044082878.1), and Gallus (GenBank accession no. NP_990113.1). The NRP1 residues at positions 35, 46, and 90 are marked in blue triangles. (**B**) Surface potential diagram of interface zone of NRP1s. The structural superposition of the NRP1 region 26–141 from human (yellow, PDB code 7m0r), rhesus (violet), hamster (purple), mouse (gray), ferret (cyan), mink (red), and chickens (green). The NRP1 structures of chickens, rhesus, hamster, ferret, and mink were modeled using the homology models of human NRP1 (PDB code 7m0r) as the template by SWISS-MODEL (https://swissmodel.expasy.org/). The three key differential residues of NRP1 interacting with the spike protein of SARS-CoV-2 are represented by yellow sticks in the structural superposition; the black dash line circled key residues in the potential surface diagram. The electrostatic potential color range is -/+5

**Fig. 3:**
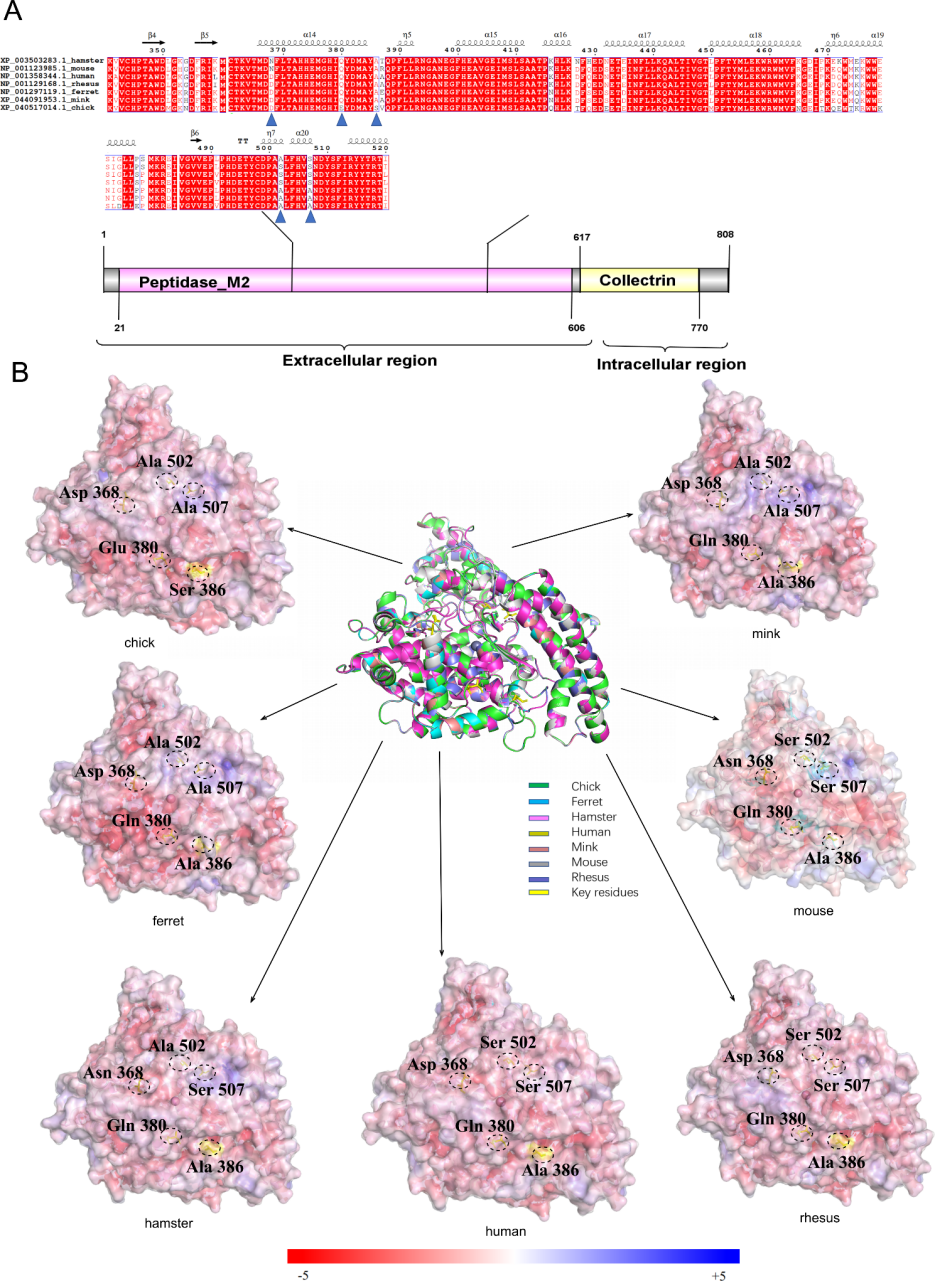
Alignment and surface potential analysis of crucial amino acids in ACE2 proteins. (**A**) Comparative analysis of the residues of ACE2s at the interface binding to the spike protein of SARS-CoV-2 from human (GenBank accession no. NP_001358344.1), rhesus (GenBank accession no. NP_001129168.1), hamster (GenBank accession no. XP_003503283.1), mouse (GenBank accession no. NP_001123985.1), ferret (GenBank accession no. NP_001297119.1), mink (GenBank accession no. XP_044091953), and chickens (GenBank accession no. XP_040517014.1). The ACE2 residues at positions 368, 380, 386, 502, and 507 are marked in blue triangles. (**B**) Surface potential diagram of interface zone of ACE2s. The structural superposition of the ACE2 region 366–520 from human (yellow, PDB code 6m18), rhesus (violet), hamster (purple), mouse (gray, UniProt entry Q0093), ferret (cyan), mink (red), and chickens (green). The ACE2 structures of chickens, rhesus, hamster, ferret, and mink were modeled using the homology models of human ACE2 (PDB code 6m18) as the templates by SWISS-MODEL (https://swis-smodel.expasy.org/). The five key differential residues of ACE2 interacting with the spike protein of SARS-CoV-2 are represented by yellow sticks in the structural superposition, the black dash line indicates the key residues in the potential surface diagram. The electrostatic potential color range is -/+5

As shown in Fig. 1A, the key residues of chickens AXL are highly similar to those of ferrets and minks infected by SARS-Cov-2 [18]. According to the human AXL-S NTD complex, His68, He75, Phe120, Gly122, and His123 are located in the interface and interact with S NTD (Fig. 1B). As shown in Fig. 2A, the three key residues of chickens NRP1 are different from the corresponding residues of ferrets, mink, and humans, and only one residue is consistent. Human NRP1-S CendR complex, Ser35, His46, and Phe90 are located in the interface and interact with S CendR (Fig. 2B). And in Fig. 3A, the key residues of chickens ACE2 are also highly similar to those of ferrets and minks.The structure of the human ACE2-S RBD complex, Asp368, Gln380, Ala386, Ser502, and Ser507 is located in the interface and interacts with S RBD (Fig. 3B).

In addition, the affinity characteristics of chickens AXL were similar to those of human AXL residues. L61H and E113F substitution of AXL may increase the affinity, V68I mutation does not disrupt the formation of hydrogen bonds, the residue at G115 had no mutation, and G116H substitution in hamster, mouse, ferret, and rhesus leads to new interactions on the interface, which might have little effect on the interfacial binding properties. The distribution of electrostatic potential indicated that the AXL-S interface pattern between chickens, ferret, mink, rhesus, and human is more identical than that of hamster and mouse. In these sites of NRP1, the residue at the S35 position is not mutated, but the affinity may decrease due to the Q46E mutation that disrupts the original hydrogen bond formation. The substitution of I90F in the hamster, mouse, ferret, and rhesus leads to new interactions at the interface, which might enhance the binding of the interface. The distribution of electrostatic potential indicated that the NRP1-S interface pattern between chickens and mouse is more identical. In these sites of ACE2, the residue at position D368 was not mutated and had no effect on interface binding; E380Q and S386A mutations do not disrupt the formation of hydrogen bonds. A502S and A507S substitutions in ferret and mink and A507S substitution in chickens, hamster, and mouse lead to new interactions at the interface, which might weaken interfacial binding. The distribution of electrostatic potential indicated that the ACE2-S interface pattern between chickens and ferret, mink, and hamster is more identical than that of rhesus. Therefore, the structural difference of AXL, ACE2 between different species indicated that chickens AXL might exhibit binding affinity with S NTD similar to that of mink and ferret. However, the structural difference of NRP1 between different species indicated that chickens NRP1 might exhibit low affinity to S CendR, which is different from that of species that are sensitive to coronavirus disease-2019 (COVID-19).

### Key residues in the receptor-S RBD/NTD complexes

**Fig. 4:**
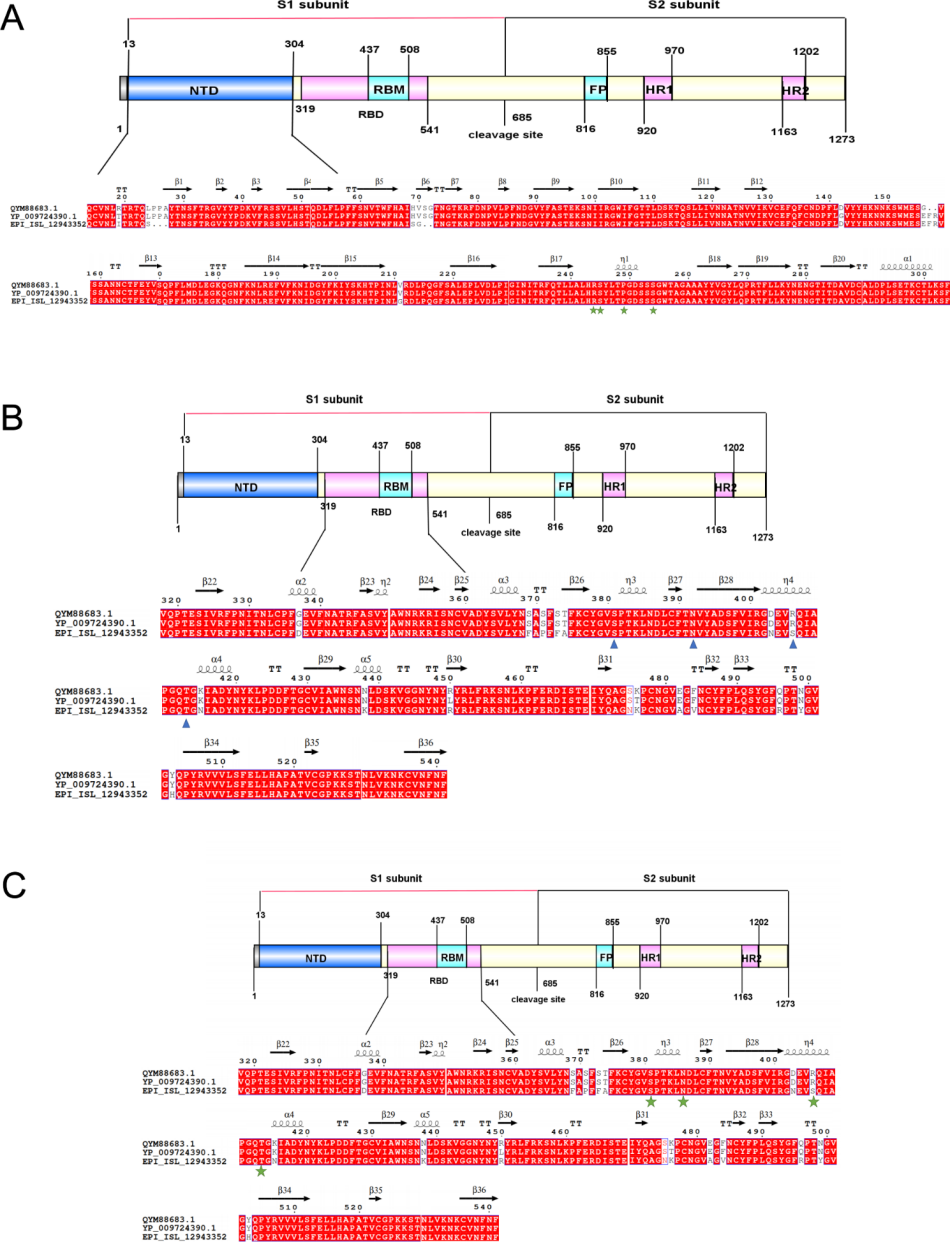
Sequence alignment of spike protein from three SARS-CoV-2 strains, including Wuhan-Hu-1 (GenBank accession no.YP_009724390.1), Delta variant (GenBank accession no. QYM88683.1), and Omicron variant (GenBank accession no. EPI_ISL_12943352). The critical residues in NTD of S interacting with AXL (**A**), CendR of S interacting with NRP1 (**B**), and RBD of S interacting with ACE2 (**C**) are marked with green stars or blue triangles

As an RNA virus, SARS-CoV-2 is prone to mutation. In the process of transmission, a dominant strain is formed gradually, stronger variants are screened, and the newly emerged Omicron and Delta variants have higher affinity and transmission speed than the original strain [19]. In order to elucidate the influence of certain changes in the spike protein of different SARS-CoV-2 variants, AA sequence was compared and analyzed. The results in Fig. 4A, B, C demonstrated that the key residues R244, S245, P249, and S254 in the AXL-S NTD complex and the key residues S381, N392, T413 in the ACE2-S RBD complex and NRP1-S CendR complex were unchanged, while only the Omicron strain was mutated to S at R406. The loss and substitution of the residues occur at the same location between the two variants that are key to the virus’s binding to receptors; nonetheless, the potential of these amino acid differences to cause transmission of SARS-CoV-2 VOCs in chickens need to be investigated further. Once SARS-CoV-2 variants have become more contagious and transmissible than previous viruses, the transmission and mutation of the zoonotic diseases between species will be more complex when these variants infect chickens.

## Discussion

In the present study, we selected AXL, NRP1, and ACE2 receptors to predict the potential susceptibility of SARS-CoV-2 VOCs to chickens. Based on the analysis using protein sequences and models, we predicted that AXL and ACE2 receptors in chickens could be used as binding sites for emerging SARS-CoV-2 variants in the future; these sites help the virus break through the barrier and cause intracellular infection in chickens. For chickens, NRP1 has less possibility to become a binding site for the virus to invade the host, but it does not rule out the possibility that it may become a potential target for the invasion of chickens cells by emerging future SARS-CoV-2 variants.

Coronavirus is an enveloped positive-stranded RNA virus that can cause respiratory and gastrointestinal symptoms, and its subfamily can be divided into four genera of *Alphacoronavirus*, *Betacoronavirus*, *Gammacoronavirus*, and *Delta coronavirus*. SARS-CoV-2 belongs to Betacoronavirus and is widely prevalent in the current world. MERS-CoV, once prevalent in the Middle East, also belongs to *Betacoronavirus*. On the other hand, chickens can be infected by *Gammacoronavirus*, showing symptoms of infectious bronchitis. Presently, turkey, guinea fowl, and pheasant can be infected by coronavirus with symptoms [20]. The current findings showed that some coronaviruses of the same genus, such as *Betacoronavirus* in dogs, cattle, and humans, exhibit high sequence consistency, suggesting genetic recombination or cross-species transmission among these viruses [21][22][23][24]. These effects are observed in the same genus of viruses, and there is no evidence of recombination between different genera of coronavirus. If coronaviruses of different genera want to recombine, the premise of recombination is that different coronaviruses infect the same cell of the same species, which has never happened before[20].

Poultry, such as chickens, turkeys, ducks, quail, and geese, are less likely to be intermediate hosts of SARS-CoV-2 VOCs in the experimental and natural infections [25]. Moreover, some studies have shown that it is difficult for SARS-CoV-2 to infect and replicate in chickens[26]. Since the emergence of the virus, SARS-CoV-2 has continually evolved, and the transmission capacity has increased. In addition, some viral receptors in vertebrates have been conserved across evolution. Based on these findings, whether the continuous mutation of the virus will increase the spike protein affinity that would make the virus break through the original species barrier and obtain a new intermediate host is yet unclear. A large number of studies are focused on the molecular dynamics of SARS-CoV-2 S protein and the protein model of receptor molecule ACE2 to confirm whether ACE2 is a receptor in different species that mediates SARS-CoV-2 entry. Some results showed that turkey’s ACE2 promotes SARS-CoV-2 entry, but some results showed that it is difficult to infect the chickens with SARS-CoV-2 VOCs[27][28]; these differences could be attributed to the diverse sequences of the proteins.

Furthermore, we conducted protein sequence alignment and interspecific protein modeling of the amino acid sequences of the discovered SARS-CoV-2 receptors AXL and NRP1, which interacted with the S proteins of SARS-CoV-2 to predict the likelihood of chickens being the intermediate host of SARS-CoV-2 VOCs. In addition to the S protein of Wuhan-Hu-1, the sequence alignment of Delta and Omicron revealed mutations at multiple sites in the Omicron variant, which might be related to its greater transmissibility than other variants.

In summary, the current study fills a gap in predicting the likelihood of chickens as a new intermediate host for SARS-CoV-2 VOCs by multiple SARS-CoV-2 receptors and enriches the current research on human-animal transmission. However, not all possible host factors for SARS-CoV-2 have been analyzed in this study because these predicted receptors have not been substantiated experimentally [28]. Additionally, we are currently conducting monitoring and experimental research on the susceptibility of emerging SARS-CoV-2 VOC to chickens, and the relevant results will be submitted in subsequent research reports.

Due to the large number, wide distribution, and close contact of humans with poultry animals, the possibility of infection with SARS-CoV-2 VOCs in chickens poses a significant threat to public health globally. The human-chickens, chickens-to-human cross-species transmission may complicate the cumulative mutation of the virus, causing an epidemic in humans. Also, SARS-CoV-2 VOCs crossing the species barrier to infect the chickens provides realistic conditions for the recombination between the diverse genera of coronavirus, thereby giving rise to further complex variants. Therefore, the current results raise the possibility of SARS-CoV-2 VOCs infection in chickens, suggesting that these birds may be the potential hosts for cross-species transmission of SARS-CoV-2, helping humans take proactive measures to respond to the next pandemic.

## Data Availability

The datasets used and/or analysed during the current study are available from the corresponding author on reasonable request.

## Abbreviations

AXL: Tyrosine kinase receptor UFO
NRP1: Neuropilin 1
ACE2: Angiotensin-converting enzyme 2
AA: Amino acid
NTD: N-terminal domain
RBD: Receptor-binding domain
SARS-Cov-2: Severe acute respiratory syndrome coronavirus 2
PDCoV: Porcine Delta-coronavirus Diease
S: Spike
COVID-19: coronavirus disease-2019
CendR: C-end Rule
